# Effects of soluble milk protein or casein supplementation on muscle fatigue following resistance training program: a randomized, double-blind, and placebo-controlled study

**DOI:** 10.1186/1550-2783-11-36

**Published:** 2014-07-11

**Authors:** Nicolas Babault, Gaëlle Deley, Pascale Le Ruyet, François Morgan, François André Allaert

**Affiliations:** 1 National Institute for health and medical research (INSERM), unit 1093, Cognition, Action and sensorimotor plasticity, Dijon, France; 2 Centre for Performance Expertise, UFR STAPS, Dijon, France; 3 Lactalis R&D, Retiers, France; 4 Chair of Medical Evaluation ESC, Dijon, France; 5 CEN Nutriment, Dijon, France; 6 Faculté des Sciences du Sport, Université de Bourgogne, BP 27877, 21078 Dijon, Cedex, France

**Keywords:** Muscle power, Endurance, Muscle thickness, Branched-chain amino acids

## Abstract

**Background:**

The effects of protein supplementation on muscle thickness, strength and fatigue seem largely dependent on its composition. The current study compared the effects of soluble milk protein, micellar casein, and a placebo on strength and fatigue during and after a resistance training program.

**Methods:**

Sixty-eight physically active men participated in this randomized controlled trial and underwent 10 weeks of lower-body resistance training. Participants were randomly assigned to the Placebo (PLA), Soluble Milk Protein (SMP, with fast digestion rate) or Micellar Casein (MC, with slow digestion rate) group. During the 10-week training period, participants were instructed to take 30 g of the placebo or protein twice a day, or three times on training days. Tests were performed on quadriceps muscles at inclusion (PRE), after 4 weeks (MID) and after 10 weeks (POST) of training. They included muscle endurance (maximum number of repetitions during leg extensions using 70% of the individual maximal load), fatigue (decrease in muscle power after the endurance test), strength, power and muscle thickness.

**Results:**

Muscle fatigue was significantly lower (P < 0.05) in the SMP group at MID and POST (-326.8 ± 114.1 W and -296.6 ± 130.1 W, respectively) as compared with PLA (-439.2 ± 153.9 W and -479.2 ± 138.1 W, respectively) and MC (-415.1 ± 165.1 W and -413.7 ± 139.4 W, respectively). Increases in maximal muscle power, strength, endurance and thickness were not statistically different between groups.

**Conclusions:**

The present study demonstrated that protein composition has a large influence on muscular performance after prolonged resistance training. More specifically, as compared with placebo or micellar casein, soluble milk protein (fast digestible) appeared to significantly reduce muscle fatigue induced by intense resistance exercise.

## Background

Resistance training simultaneously stimulates catabolism and anabolism in active muscle fibers. The difference between these mechanisms is called net protein balance. When positive, the net protein balance favors increases in muscle mass, i.e., muscle hypertrophy. The effect of resistance training on net protein balance can persist up to 48 h [1]. In addition, any nutritional modification that could increase protein accretion in the muscle would maximize resistance training effects by enhancing muscle anabolism. In particular, it has now been well demonstrated that protein consumption after exercise shifts the balance in favor of muscle protein synthesis [2].

Composition of supplements may play a key role in influencing net protein balance since previous studies have revealed that only essential amino acids could stimulate muscle protein synthesis [3]. Furthermore, protein type, and not simply its amino acid composition, can differentially modulate protein synthesis depending on digestion kinetics. For instance, milk contains two protein fractions, soluble proteins and micellar casein, with rapid and slow digestion rates, respectively [4]. As a consequence, muscle protein synthesis has been shown to be greater with soluble proteins such as whey when compared with casein [5].

In addition to increases in muscle mass, functional adaptations, such as strength increases, are also obtained after essential amino acid (EAA) supplementation [6]. For example, Vieillevoye et al. [7] found increases in lower body strength with EAA supplementation while no modification was obtained with placebo. Protein supplementation may also influence muscle fatigue. Indeed, previous authors [8] have reported an attenuation of fatigue during repeated bouts of dynamic contractions after four weeks of protein supplementation as a result of an increased muscle buffering capacity during endurance exercise. Other amino acids, such as branched-chain amino acids (BCAA; leucine, isoleucine and valine), might also reduce fatigue by lowering perceived exertion and favoring mental performance during prolonged exercise [9,10]. Taken altogether, these results seem to suggest that the effects of protein supplementation are largely dependent on their composition, and it can be hypothesized that supplementation with a rapidly-digesting protein would be more efficient to improve both strength and resistance to fatigue. Given that different amino acid compositions could easily be obtained from protein milk extraction, the aim of the present study was to compare the effects of two different formulations of milk protein supplementation, one fast (a soluble milk protein) and one slow (micellar casein), on muscle performance (endurance, fatigue, strength and power) during and after a 10-week resistance training program.

## Methods

### Participants

A total of 68 male participants were recruited for the study. All were practicing two to six hours of physical activity per week (<3 sessions a week). None were engaged in any physical activity aimed at increasing the size and strength of knee extensor muscles. All were healthy and free of injury in the three months preceding the study. The study excluded subjects who had previously received treatment with corticoids. Participants who were currently taking any dietary supplement, sports drink, or functional food intended to enhance performance or muscle mass, or had taken any of these in the previous month, were also excluded. Moreover, subjects with known hypersensitivity to any of the constituents of the products under study (milk protein or lactose) were excluded. Throughout the study, subjects maintained their usual training routines and diets. All gave their written informed consent after being told about the experimental procedure. The study was conducted in accordance with the Helsinki Declaration and was approved by the local ethics committee (East I, number: 2011–38, 4 October 2011, AFSSAPS number: 2011-A00789-32).

After inclusion, participants were randomly divided into three experimental groups: 24 in the Placebo group (PLA), 22 in the Micellar protein group (MC), and 22 in the soluble milk protein group (SMP). Balanced randomization was made by blocks of four. The randomization code was not made available to anyone involved in conducting or evaluating the study and was released after the blind review and the freezing of the final database. The sample size was calculated *a priori* using Nquery Advisor software (ver. 6.01, Statistical solutions Ltd, Cork, Ireland) based on the primary criterion (muscle endurance) and allowing for a power > 90%. This statistical analysis indicated a minimum of 22 participants per experimental group.

### Experimental procedure

**Figure 1:**
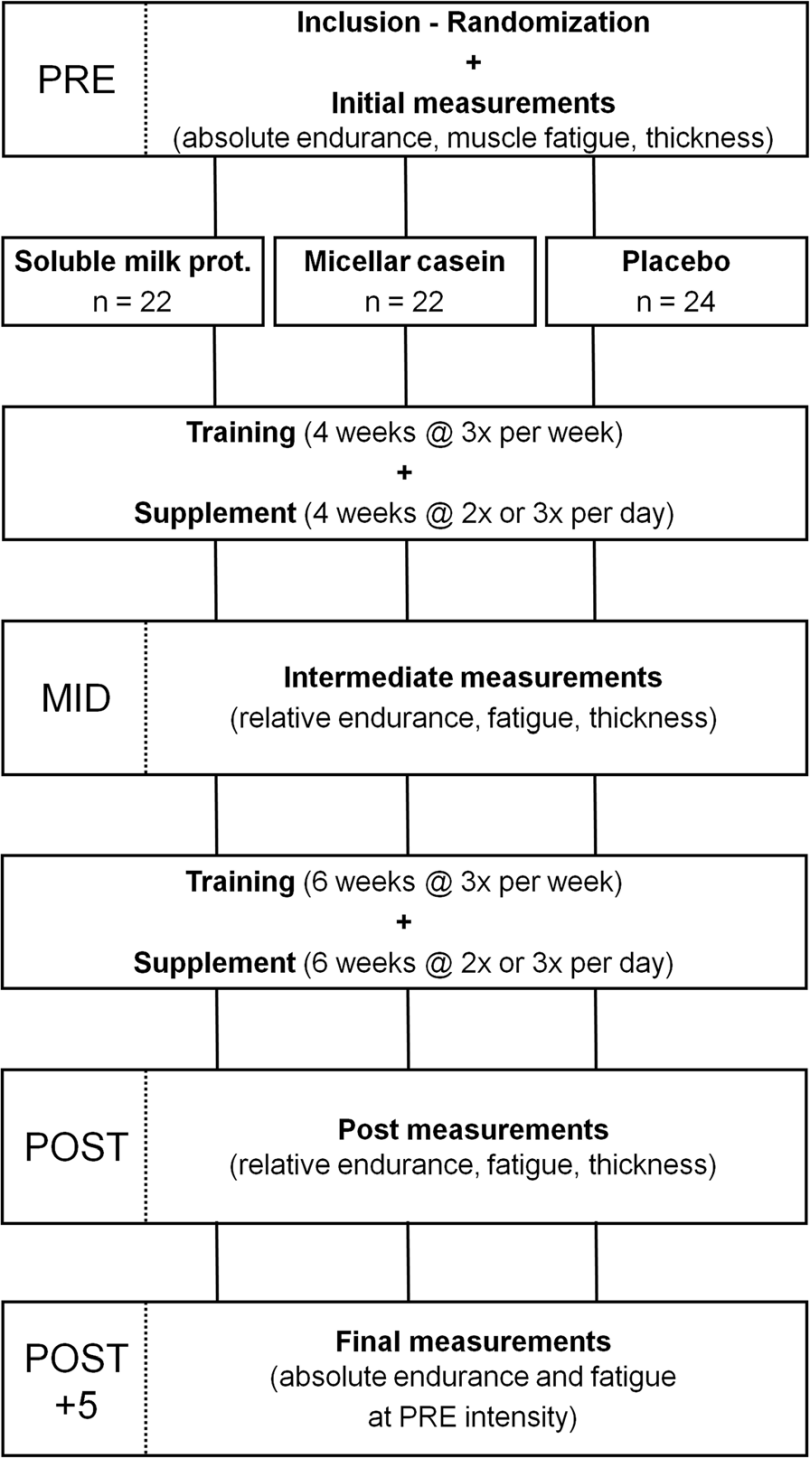
Illustration of the experimental procedure.

The primary objective of this randomized, double-blind study, conducted with parallel arms, was to evaluate the effects of different milk protein supplements on muscle endurance and fatigue following a resistance training program. A soluble milk protein beverage was compared with a micellar casein beverage and placebo. Body composition, knee extensor muscle thickness, maximal strength, power and perceived exertion were also determined.The experiment involved four testing sessions: one at inclusion (PRE), one at the middle of the training program (after 28 days; MID) and two at the end of the 10 weeks training program (POST and POST + 5) (Figure 1). Testing sessions were conducted on non-training days and always at the same time of day for a given subject. PRE, MID and POST sessions included measurements of (i) right vastus lateralis muscle thickness using ultrasonography, (ii) lower limb muscle power during vertical jumps, (iii) maximal strength on a leg extension machine (one repetition maximum, 1-RM), (iv) muscle power on the same leg extension machine at 70% of the 1-RM, (v) muscle endurance during an all-out test (maximum number of repetitions performed with a load corresponding to 70% of the session’s 1-RM) and (vi) recovery, as determined by muscle power, immediately, 30 min and 60 min after the endurance test. Tests were always performed in the aforementioned order after a standardized warm-up. Warm-up consisted of light pedaling followed by sub-maximal voluntary contractions and vertical jumps in order to familiarize subjects with testing procedures. The POST + 5 session only consisted of an all-out endurance test performed with the same load as the one used at PRE, followed by recovery power measurements.

All subjects followed the same 10-week resistance training routines, three times per week with a rest day between sessions. The program was based on three exercises involving knee extensors (knee extension machine and horizontal leg press) and knee flexors (hamstring curl machine) (Technogym, Gambettola, Italy). Throughout the training program, sets number, repetition number and recovery between sets varied: from the first to seventh week, sets increased from three to five and repetition maximum (RM) number also increased from eight to 15. During weeks eight and nine, training consisted of four sets of 20 RM. During the last week, training consisted of five sets of six RM. Recovery beteween sets was 1–2 minutes. The load used for each exercise was regularly adapted depending on the 1-RM evaluated every two weeks. All training sessions were supervised by specialized teachers.

### Dietary supplementation

The three products under study were presented as 30-g sachets to be diluted in 200 mL water. Once the powders were diluted in cold water, drinks were of identical appearance, texture, taste, and all were isoenergetic (120 kcal per 30 g powder). Products were taken during the 10-weeks training period. On non-training days, protein intake was repeated twice, with the first in the morning after waking and the second during the afternoon. During training days, protein intake was repeated three times: in the morning and 30 min before and after the resistance training session. Protein supplementation was done either with Prolacta® or micellar casein.

Prolacta® (Lactalis, Retiers France) is a 90% soluble milk protein isolate. It is representative of native proteins in the non-casein phase of milk as it is a concentrate of native whey proteins extracted directly from skimmed cow milk by a soft membrane process (Lactalis Industry, Bourgbarré, France), and not from whey. Prolacta® is defined as a fast leucine-rich protein [4] differing from whey with a better amino acid composition. Moreover, Prolacta® has demonstrated larger postprandial protein retention than casein [11]. Each 30 g Prolacta® sachet contained 10 g of protein from Prolacta®, 10.5 g sucrose, 8.2 g maltodextrine, 0.3 g lactose and 1 g soy lecithin. Each 30 g micellar protein sachet (produced by Lactalis, Retiers France) contained 10 g of Micellar Casein, 10.5 g sucrose, 7.5 g maltodextrine, 1 g lactose and 1 g soy lecithin. The amino acid composition of each product is detailed in Table 1. Placebo composition was 10.5 g sucrose and 19.5 g maltodextrine and did not contain any protein.

**Table 1:** Amino acids composition (g) for 100 g of soluble milk protein (SMP) or micellar casein (MC)

	**SMP**	**MC**
Alanine	4.77	2.84
Arginine	2.25	3.50
Aspartic acid	11.34	6.44
Cystine	3.06	0.41
Glutamic acid	17.10	21.75
Glycine	1.98	1.74
Histidine	2.00	2.70
Isoleucine	5.04	4.92
Leucine	12.00	9.05
Lysine	9.63	7.39
Methionine	2.07	3.21
Phenylalanine	3.78	4.86
Proline	4.59	10.3
Serine	4.50	5.02
Threonine	5.04	3.67
Tryptophan	2.07	1.07
Tyrosine	3.42	5.03
Valine	5.13	6.22

### Measurements

#### Muscle thickness

The right vastus lateralis muscle thickness was measured in real time using an ultrasound machine (AU5, Esaote Biomedica, Florence, Italy) coupled to a 50 mm probe at a 7.5 MHz frequency. Subjects were in the supine position with the knee flexed at a 45° angle. The probe was placed perpendicular to the skin surface in the middle of vastus lateralis muscle, i.e., 39% of thigh length measured from the superior border of the patella to the anterior superior iliac spine [12]. Thickness was calculated as the distance between superficial and deep aponeuroses measured at the ends and middle of each 3.8-cm-wide sonograph. Three images were independently obtained. The average value of these nine measures was calculated. Probe placement was carefully noted for reproduction during the other test sessions. Also, body composition (body weight, percent body fat) was quantified using a bio-impedance scale Fitness scale 7850 (Soehnle GmbH, Murrhardt, Germany).

#### Muscle strength

Subjects were seated on a leg extension machine (Multiform, La Roque d’Anthéron, France) with a 100° hip angle. The knee rotation axis was aligned with the machine rotation axis. The 1-RM was first determined after a standardized warm-up using five different loads and individually adjusted increments. Subjects were requested to lift each load only once. One minute of rest was permitted between trials. Care was taken to lift the load with a full range of motion (~100°). Range of motion was controlled using an electronic goniometer (Myotest, Sion, Switzerland).

#### Muscle endurance

Subjects were asked to lift a load corresponding to 70% of their 1-RM as many times as possible over a 100° range of motion. The test was stopped when subjects were unable to lift the load over a 80° range of motion during two consecutive repetitions. The number of repetitions was identified as subjects’ “relative endurance” (PRE, MID and POST). Immediately after all endurance tests, the rating of perceived exertion was determined using a Borg scale [13]. The endurance test procedure was repeated five days after the end of the experimental period (POST + 5), but using the same load as the one used at inclusion (here called “absolute endurance”).

#### Muscle fatigue and recovery

Muscle power and vertical jump performance were measured just before, immediately after, 30 min after and 60 min after the endurance tests, to evaluate muscle fatigue and recovery. Muscle power was measured using a linear encoder (Globus, Codogne, Italy) on the leg extension machine with the same load as during the endurance test (70% of the 1-RM). Subjects were requested to lift the load as fast as possible (3 trials) throughout the 100° range of motion. The linear encoder measured the vertical velocity of the load being lifted and allowed measurements of muscle power during the entire range of motion. Peak power of the best repetition was considered for analyses. Immediately after muscle power assessments, subjects performed two counter movement jumps on an Optojump system (Optojump, Microgate, Bolzano, Italy), starting from a standing position, then squatting down to a 90° knee angle and extending the knees in one continuous movement. During these jumps, arms were kept close to the hips to minimize their contribution. The best jump height was retained for analyses.

### Statistical analyses

Quantitative variables were presented as mean values and standard deviations (SD). Values were tested using a repeated measures analysis of variance. Groups (PLA, SMP and MC) were used as independent variables and time (PRE, MID, POST or POST + 5) was used as the dependent variable. A sensitivity analysis was also conducted and considered subjects with a muscle thickness at inclusion <22 mm (median value of study sample). Thirteen subjects were considered in both PLA and MC groups and 8 for SMP. In the case of significant main effects or interactions, Scheffé post-hoc tests were conducted. Qualitative variables (supplementation compliance or adverse effects) were presented as absolute and relative frequencies and were tested by using a Chi square test. Statistics were conducted using SAS software (Ver. 9.2, SAS Institute, Inc., Cary, NC, USA). *P* < 0.05 was taken as the level of statistical significance for all procedures.

## Results

### General observations

Initial values measured at PRE revealed comparable groups (Table 2). During the experimental protocol, compliance was evaluated by the percentage of products returned by subjects. The results show high average and comparable compliance between groups: 90.5%, 91.5% and 89.4% for PLA, MC and SMP groups, respectively (*P* = 0.769). In addition, tolerance to the three products under study was good and comparable in terms of frequency and nature. Of the 68 subjects who took products at least once, three subjects presented adverse events in each group. None of these events were due to the supplements ingested, but rather to personal convenience or injuries.

**Table 2:** Main subjects’ characteristics at inclusion (PRE)

	**PLA**	**MC**	**SMP**	**ANOVA**
Age (years)	22.0 ± 3.9	22.2 ± 3.9	22.5 ± 4.1	*P =* 0.912
BMI (Kg/m^2^)	23.4 ± 3.7	23.7 ± 3.5	22.7 ± 2.4	*P*-0.546
Physical activity (hours/week)	4.1 ± 2.2	5.1 ± 2.4	5.4 ± 3.0	*P =* 0.193
Vastus lateralis thickness (mm)	21.0 ± 3.2	21.5 ± 3.1	21.5 ± 3.5	*P =* 0.845
Counter movement jump (cm)	32.4 ± 6.0	32.2 ± 4.1	32.6 ± 4.5	*P =* 0.971
1-RM (kg)	87.7 ± 16.7	86.6 ± 17.7	83.6 ± 16.0	*P =* 0.702
Number of repetitions	16.5 ± 4.0	16.8 ± 3.4	15.5 ± 3.9	*P =* 0.527
Perceived exertion	13.9 ± 2.1	14.3 ± 2.2	14.0 ± 1.9	*P =* 0.820

Data are mean values ± SD. PLA: placebo; MC: micellar casein; SMP: soluble milk protein; BMI: body mass index; 1-RM: maximum load lifted by leg extension.

### Muscle endurance

Absolute muscle endurance (Table 3), evaluated as the number of repetitions at 70% of the initial 1-RM, increased in all groups (+90.8 ± 58.8%, +81.2 ± 51.0% and +73.7 ± 32.2% for SMP, MC and PLA, respectively) with no significant difference between groups (*P* = 0.492). However, when subjects with small muscle thickness were considered separately (thickness < 22 mm, sensitivity study), increases reached the significant level between SMP and PLA (+103.0 ± 61.1% and +65.0 ± 33.9%, respectively, *P* < 0.05). Similar results were obtained for relative endurance measured POST with a significant time effect but no differences between groups (Table 3). For all subjects, the 1-RM load was significantly increased after training (Table 3). No significant interaction was obtained for the rating of perceived exertion.

**Table 3:** Changes of the main outcomes during and at the end of the experimental procedure

	**PRE**	**MID**	**POST**	**POST + 5**
Muscle endurance (number of repetitions)*				
SMP	15.5 ± 3.9	17.3 ± 4.7	19.2 ± 7.1	25.2 ± 13.7
MC	16.8 ± 3.4	19.3 ± 4.6	21.3 ± 6.0	25.8 ± 9.5
PLA	16.5 ± 4.0	18.5 ± 6.0	20.5 ± 7.0	25.2 ± 10.4
1-RM (kg)*				
SMP	83.5 ± 16.0	91.3 ± 13.7	96.1 ± 12.4	-
MC	86.6 ± 17.7	98.9 ± 18.2	98.9 ± 17.4	-
PLA	87.7 ± 16.7	100.3 ± 13.3	104.8 ± 11.3	-
Muscle thickness (mm)*				
SMP	21.5 ± 3.5	-	22.2 ± 3.5	-
MC	21.8 ± 3.1	-	22.1 ± 3.1	-
PLA	21.0 ± 3.2	-	22.0 ± 3.6	-
% body fat				
SMP	8.6 ± 2.2	8.5 ± 2.5	8.9 ± 2.6	-
MC	9.4 ± 4.3	9.8 ± 5.1	10.0 ± 4.6	-
PLA	9.9 ± 4.6	9.9 ± 4.5	10.3 ± 5.0	-
% body fat free mass				
SMP	48.2 ± 2.0	47.9 ± 2.0	47.9 ± 2.3	-
MC	47.1 ± 3.1	46.9 ± 3.5	46.7 ± 3.2	-
PLA	46.9 ± 3.4	47.4 ± 4.0	46.7 ± 3.4	-
Body mass (kg)				
SMP	70.8 ± 8.7	72.5 ± 8.1	72.2 ± 8.9	-
MC	77.7 ± 14.7	79.0 ± 15.1	79.2 ± 15.3	-
PLA	76.6 ± 13.3	77.7 ± 12.7	78.3 ± 13.2	-

Data are mean values ± SD. PLA: placebo; MC: micellar casein; SMP: soluble milk protein; BMI: body mass index; 1-RM: maximum load lifted by leg extension. *: significant effect (*P <* 0.05).

### Muscle fatigue and recovery

**Figure 2:**
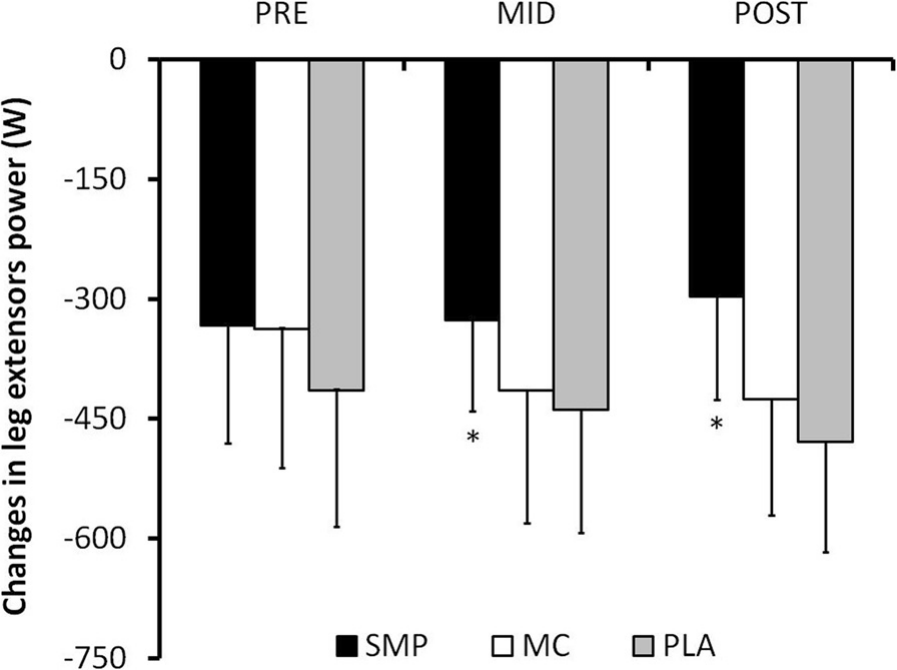
**Muscle power decrease during the endurance test.** Mean (±SD) decreases in muscle power during the endurance test before the training period (PRE), after 4 weeks training (MID) and at the end of the training (POST). *: significant differences between groups (*P <* 0.05).

Muscle fatigue, i.e. power reduction during the endurance test, was similar between groups at PRE. This decrease in power was significantly lower for SMP as compared with MC and PLA after four weeks (-326.8 ± 114.1 W, -415.1 ± 165.1 W and -439.2 ± 153.9 W, respectively; *P* = 0.0483) and after 10 weeks of training (-296.6 ± 130.1 W, -413.7 ± 139.4 W and -479.2 ± 138.1 W, respectively; *P* = 0.0004) (Figure 2). Accordingly, when comparing PRE and POST, fatigue was significantly reduced for SMP while it increased for MC and PLA (-35.9 ± 133.5 W, +89.3 ± 97.7 W and +64.8 ± 154.6 W, respectively; *P =* 0.0125).

When considering PRE and POST + 5, slight between-groups differences were registered for counter movement jump height. For SMP, counter movement jump height measured immediately after the endurance test slightly increased while it decreased for MC and PLA (+0.9 ± 4.6 cm, -1.6 ± 2.3 cm and -0.7 ± 2.8 cm, respectively; *P =* 0.0686). A small difference was obtained for counter movement jump at POST + 5. As compared with baseline, 30 min after the endurance test, counter movement jump height significantly increased in SMP and PLA while it remained lower for MC (+1.9 ± 5.1 cm, +0.4 ± 2.1 cm and -0.6 ± 2.8 cm, respectively; *P =* 0.0965).

### Muscle thickness

Muscle thickness and body composition changes are indicated in Table 3. While a significant increase in muscle thickness was obtained with time, no difference was registered between groups. Also, no significant difference was noticed for body mass, percent body fat or percent body fat free mass (Table 3).

## Discussion

The present study aimed to test the hypothesis that supplementation with milk proteins of varying compositions, used in combination with resistance training, would have different effects on physical performance. The main results revealed enhanced muscle endurance (i.e., number of repetitions), reduced fatigue (i.e., muscle power loss) and slightly enhanced recovery (i.e., vertical jump height) with a supplement composed of fast-digesting protein (soluble milk protein) as compared with a slow-digesting protein (micellar casein) or with a placebo. It should be noted that differences between groups were larger when subjects with lower initial muscle thickness were considered.

Increased muscle endurance and reduced muscle fatigue were observed in the group with fast protein supplementation. Similar results have previously been obtained with acute intakes [14-16] and with chronic diets [17], while others failed to register any effects [18,19]. Three main mechanisms could be involved in the observed enhancement of muscle endurance and fatigue. The first may be related to glucose and glycogen availability. However, Falavigna et al. [17] concluded that chronic BCAA supplementation has no effect on glucose metabolism and could therefore be excluded. The second may originate from reduced exercise-induced muscle protein degradation. Indeed, Hoffman et al. [14] reported lower creatine kinase activity following resistance exercise with acute protein supplementation. Moreover, they also registered improved recovery 24 and 48 h post exercise. Such results may be attributable to an enhanced repair process through reduced protein breakdown and increased protein synthesis [15]. However, due to the widely different experimental designs, such a mechanism is unlikely when considering our muscle endurance results. A third mechanism, related to a reduced central fatigue, could also be possible. Indeed, BCAA intake may reduce the plasma ratio of free tryptophan/BCAA and therefore the transport of tryptophan into the brain. This would reduce the synthesis, concentration and release of 5-hydroxytryptamine that is directly related to the development of fatigue and to the consequent reduction of performance [10]. In humans, such an effect has mostly been evidenced by a reduced feeling of fatigue during exercise and also during cognitive tasks such as short-term memory [9]. In the present study, no difference was registered in perceived exertion at the end of the muscle endurance test but the number of repetitions was increased with SMP. Therefore, our results are in general accordance with the literature. In addition, SMP induced a reduction in muscle fatigue. Although unclear, such an increase in muscle endurance could be attributed to the protein composition and digestibility. Indeed, with rapid digestibility, whey has been shown to induce a transient and more pronounced rise in whole body protein synthesis than casein [1,4]. Therefore, it could be speculated that soluble milk protein, tested here, may reduce central fatigue as a result of this enhanced whole body protein synthesis. Moreover, the amino acid composition of the two tested protein beverages is different, which could have a significant impact on the reported outcomes [5]. For example, cysteine content is seven times greater in SMP as compared to MC. It is well known that cystine enhances glutathione synthesis [20], an endogenous muscle antioxidant. Using quite similar cysteine contents as in our study while comparing whey vs. casein, authors demonstrated the positive effects of daily cysteine supplementation on the augmentation of antioxidant defense and anaerobic cycling performance [21]. Similar positive results were obtained for fatigue and performance during various exercises [22,23] with increases in cystine, cysteine, and glutathione content as a result of N-acetylcysteine ingestion [22]. Therefore, beverages with various amino acid content and not only BCAA or leucine are beneficial during prolonged exercise.

Muscle strength, power and thickness, although improved after the experimental period, were not different between groups. Such results are quite surprising since both protein beverages should have enhanced muscle thickness and muscle strength increases as compared with the placebo. Indeed, protein and more particularly BCAA are well known to increase muscle protein synthesis. For example, leucine plays a major role in muscle protein synthesis [24-26] through the stimulation of the mammalian target of rapamycin signaling pathway [27]. The lack of differences in muscle thickness and strength between groups remains unclear but could be attributed to several factors such as the supplement characteristics, training type and training status.

Protein was supplemented twice on non-training days and three times on training days. On training days, protein was ingested before and after the exercise session. Although debated, such timing may appear as one of the most effective nutrient timing strategies for muscle protein synthesis [26]. As compared to a morning/evening intake group, Cribb and Hayes [28] observed larger muscle mass increases with protein intake before and after training. The best stimulus for protein synthesis appeared to be with protein feeding in close proximity to training sessions [29,30], with feeding recommended within the first two hours postexercise [31-33].

Protein quantity was 20 or 30 g.day^-1^ (non-training and training days, respectively) with 10 g before and 10 g after resistance training sessions. Prima facie, these doses seem unlikely to be responsible for the lack of difference between groups for muscle thickness or muscle strength. However, associated with the timing, a dose–response relationship for protein synthesis is generally obtained. For example, although the contents of EAA and BCAA used in the current study would be sufficient to stimulate the mammalian target of rapamycin signaling pathway, a dose twice as large seems more efficient [34]. In a recent review [35], Phillips recommended the ingestion of 20 g of high quality protein immediately after exercise to maximally stimulate protein synthesis; and several authors reported that consumption of 8 to 11.5 g of EAA containing 2 to 3 g of leucine after exercise may maximize the protein synthetic response [5,36,37]. Recently, authors have demonstrated that low doses of leucine (0.75 g) may stimulate muscle protein synthesis following resistance exercise in young healthy individuals [38]. Higher doses (3 g leucine), however, maintained muscle protein synthesis longer and may be more effective for anabolism after resistance exercise [38]. In our study, the suboptimal doses ingested after exercise might therefore have reduced the potential protein effects and the potential differences between groups for muscle strength and thickness. This dose–response relationship may also explain differences between groups for muscle endurance and fatigue. For example, Falavigna et al. [17] registered increased or reduced time to exhaustion during prolonged swimming in rats with low or high BCAA diets. Intakes should therefore be adequately chosen to obtain optimal adaptations.

It should be remembered that training and supplementation effects are potentiated in subjects exhibiting lower muscle thickness at inclusion. Such a result is not surprising since training is well known to have larger effects in untrained subjects. For example, greater increases in muscle cross-sectional area have been reported in subjects who had not previously engaged in resistance training in comparison with more accustomed subjects [39]. The effects of amino acids supply may also depend on training status, since greater disturbances in protein turnover (protein synthesis and degradation) are obtained following training in novice than in experienced athletes [40]. Moreover, the expected increase in protein synthesis following exercise appears to be smaller and shorter in trained athletes as compared with untrained subjects [41,42]. Thus, training status may influence muscle performance. Indeed, Vieillevoye et al. [7] found increases in lower body strength with an essential amino acid supplement while no modification was obtained with placebo. Surprisingly, in the same study, strength was similarly enhanced in both groups for the upper body. These authors concluded that supplementation and training adaptations seem to depend on the initial training status; the weaker the subjects, the larger the effect of protein supplementation on muscle strength. Moreover, the present study was conducted in physically active males. Hence, it is possible to speculate that, with untrained participants, differences between groups might have been revealed. Furthermore, a plateau, or ‘ceiling effect’ , of the adaptive responses to training is generally observed for strength gains and the muscle protein synthetic response [40,43]. Hence, protein requirements and training stimulus are affected by training status and duration. For instance, greater protein intakes are required during the early stages of intensive bodybuilding training and more particularly in novices [44]. Modification of the training program might also have exacerbated differences between groups for all studied parameters. Training volume [45] concomitant with the load used in terms of 1-RM’s percentage [46] are possible parameters.

## Conclusions

In conclusion, the present experiment demonstrated that protein supplementation may enhance possible adaptations induced by resistance training. Our results suggest that the effects of protein supplementation depend on the protein composition. More specifically, it appears that soluble milk protein is particularly efficient to improve resistance to fatigue. Therefore, supplementation with soluble milk protein may be recommended in combination with to resistance training.

## Abbreviations

BCAA: Branched-chain amino acids; MC: Micellar casein; MID: Testing in the middle; PLA: Placebo; PRE: Testing at inclusion; POST: Testing at the end; RM: Repetition maximum; SMP: Soluble milk protein.

## Competing interests

Lactalis Recherche et Développement provided financial support to conduct the study. The funders have no role in data collection and analysis or preparation of the manuscript. PLR and FM, two authors, have an affiliation (employment) to the commercial funders of this research.

## Authors’ contributions

NB (corresponding author) was responsible for the study design, the execution of the measurements and the writing of the manuscript. GD participated in the study design and the writing of the manuscript. PLR and FM participated in the study design. FAA participated in the study design, the statistical analysis and the writing of the manuscript. All authors read and approved the final manuscript.
